# International multicentre evaluation of a new anti‐idiotypic anti‐daratumumab for resolving pre‐transfusion interferences

**DOI:** 10.1111/vox.70269

**Published:** 2026-04-20

**Authors:** Arnaud Reggiani, Sofia Lejon Crottet, Sophie Waldvogel, Charlotte Engström, Serelina Coluzzi, Antonella Matteocci, Janis R. Hamilton, Levent Tufan Kumas, Karen M. K. de Vooght, Jill R. Storry, Jennylyn Flores, Núria Nogués, Carlos Cotorruelo, Molly Rutherford, Eveline B. Nilsen, Monika Pelc‐Kłopotowska, Jolanta Korzeniowska, Alessandra Baffa, Inna Sareneva, Nor Hafizah Ahmad, Katarzyna Szczudlo, Athif Rahman, Laser Sanal, Lise Jørgensen, Karolina Majewska Rochowiak, Monika Salwierz, Elisaveta Grancharova, Bojana Zivkovic, Jessica Alvarez, Mareike Riedl, Achim Knappik

**Affiliations:** ^1^ Bio‐Rad Laboratories DiaMed GmbH Cressier Switzerland; ^2^ Interregional Blood Transfusion SRC Ltd. Bern Switzerland; ^3^ Laboratoire d'immuno‐hématologie transfusionnelle Hopitaux Universitaires de Genève Genève Switzerland; ^4^ Blood Transfusion Service Zurich SRK Schlieren Switzerland; ^5^ AOU Policlinico Umberto 1‐Sapienza UOC Immunoematologia e Medicina Trasfusionale Rome Italy; ^6^ Azienda Ospedaliera San Camillo – Forlanini UOC Medicina Trasfusionale e Cellule Staminali Rome Italy; ^7^ American Red Cross Michigan Region Detroit Michigan USA; ^8^ Acıbadem Labmed İstanbul Turkey; ^9^ Central Diagnostics Laboratory UMC Utrecht Utrecht the Netherlands; ^10^ Department of Clinical Immunology and Transfusion Medicine Skåne University Hospital Lund Sweden; ^11^ Children's National Hospital Laboratory Blood Bank Washington DC USA; ^12^ Immunohematology Laboratory Banc de Sang i Teixits Barcelona Spain; ^13^ Laboratorio de Inmunohematología Universidad Nacional de Rosario Rosario Argentina; ^14^ Synnovis Analytics LLP, Guy's and St Thomas' Hospital London UK; ^15^ The Blood Bank at Vestfold Hospital Trust Tønsberg Norway; ^16^ Institute of Hematology and Transfusion Medicine Warsaw Poland; ^17^ Regionalne Centrum Krwiodawstwa i Krwiolecznictwa w Lublinie Lublin Poland; ^18^ S.C. Banca del Sangue e Immunoematologia Turin Italy; ^19^ Department of Clinical Chemistry, Diagnostic Center Helsinki University Hospital and University of Helsinki Helsinki Finland; ^20^ National Blood Centre Immunohematology Reference Laboratory Kuala Lumpur Malaysia; ^21^ Regional Blood Donation and Blood Therapy Center Katowice Poland; ^22^ Blood Transfusion Department Hammersmith Hospital London UK; ^23^ Ankara Bilkent City Hospital Transfusion Center Ankara Türkiye; ^24^ Department of Immunology and Transfusion Medicine Oslo University Hospital Oslo Norway; ^25^ Regionalne Centrum Krwiodawstwa i Krwiolecznictwa w Krakowie Kraków Poland; ^26^ Rzeszów – Regionalne Centrum Krwiodawstwa i Krwiolecznictwa Rzeszów Poland; ^27^ Center of Transfusion Hematology, Military Medical Academy Sofia Bulgaria; ^28^ UKC Maribor Maribor Slovenia; ^29^ UChicago Medicine Center for Care and Discovery Chicago Illinois USA; ^30^ Bio‐Rad AbD Serotec GmbH Neuried Germany

**Keywords:** anti‐CD38, anti‐idiotypic antibody, daratumumab, multiple myeloma, pre‐transfusion testing, RBC alloantibodies

## Abstract

**Background and Objectives:**

Daratumumab, a therapeutic human anti‐CD38 monoclonal antibody, improves multiple myeloma outcomes but interferes with pre‐transfusion testing by binding CD38 on reagent red blood cells (RBCs), potentially masking clinically significant alloantibodies. This study aimed to evaluate the effectiveness of a novel high‐affinity anti‐idiotypic anti‐daratumumab reagent in neutralizing daratumumab interference while preserving RBC alloantibody detection and ensuring compatibility with routine transfusion laboratory workflows.

**Materials and Methods:**

A non‐interventional, multicentre study across 28 transfusion laboratories in 15 countries evaluated a novel anti‐idiotypic anti‐daratumumab reagent (DaraClear). In total, 443 daratumumab‐containing plasma samples and 197 RBC alloantibody–containing samples were tested. All samples were initially tested at 10% (v/v), with stepwise escalation to 20% and 30% only if neutralization was incomplete. Neutralization efficiency, antibody detection, cross‐match resolution and workflow integration were assessed, alongside comparisons with dithiothreitol (DTT), papain, trypsin and DaraEx.

**Results:**

Daratumumab interference was neutralized in 99.5% of samples, with 86.2% resolved using the 10% protocol. Detection of 190/197 RBC antibodies (96.4%) was preserved, including weak/low‐titre antibodies. Neutralization was superior to DTT (93.3%), papain/trypsin (84.9%) and DaraEx (68.4%; all *p* < 0.0001). Titration studies confirmed efficacy across various ranges of daratumumab titres. The reagent integrated seamlessly into workflows, remained stable over time and avoided RBC modification.

**Conclusion:**

Daratumumab interference was efficiently neutralized while preserving alloantibody detection. The robust performance and workflow compatibility provide a practical solution for pre‐transfusion testing in daratumumab‐treated patients, supporting safe and timely transfusions with reduced laboratory burden.

**Highlights**

- Large multicentre validation: A novel anti‐idiotypic anti‐daratumumab reagent was successfully evaluated across 28 laboratories in 15 countries.
- High neutralization efficiency while preserving alloantibody detection: Daratumumab interference was neutralized in 99.5% of samples, preserving 96.4% of red blood cell (RBC) antibodies.
- Practical and clinically impactful: Effective across variable daratumumab titres, simple to implement, without RBC modification, and supports safe, timely transfusions with reduced laboratory workload.

## INTRODUCTION

Immunotherapies have transformed cancer treatment, shifting the paradigm from conventional chemotherapy to targeted approaches that deliver improved clinical outcomes with reduced toxicity [1, 2]. Monoclonal antibody (mAb) therapies are now central to the management of both haematological malignancies and solid tumours, demonstrating high efficacy and tolerability [3, 4].

CD38, a 46‐kDa type II transmembrane glycoprotein, is highly expressed on malignant plasma cells in multiple myeloma (MM) but only minimally on normal lymphoid and myeloid cells, making it an attractive therapeutic target [5]. Daratumumab, a human anti‐CD38 mAb, is approved by both the European Medicines Agency and the US Food and Drug Administration for MM treatment [6, 7] and is being investigated in other settings, including ABO‐incompatible stem cell transplantation, post‐transplant autoimmune haemolytic anaemia (AIHA), immune thrombocytopenia and pure red cell aplasia [8, 9, 10].

Red blood cell (RBC) transfusions remain a cornerstone of supportive care in these patients; however, alloantibody formation against RBC antigens can lead to delayed haemolytic transfusion reactions [11, 12]. Daratumumab complicates pre‐transfusion compatibility testing because CD38 expression on reagent RBCs causes pan reactivity in the indirect antiglobulin test (IAT), thereby masking underlying alloantibodies and delaying the provision of compatible blood for transfusion [13, 14, 15].

Current transfusion strategies for daratumumab‐treated patients often involve providing phenotype‐ or genotype‐matched RBCs. However, the incidence of alloimmunization in this population is lower than in other chronically transfused groups, such as patients with sickle cell disease or myelodysplastic syndromes, raising questions about the necessity of extensive matching [16, 17, 18]. A reagent that enables a return to standard pre‐transfusion protocols could improve the safety and timeliness of transfusions, reduce laboratory workload, preserve extended phenotype‐matched RBC units and allocate laboratory resources for patients with more complex clinical needs.

Existing mitigation strategies, such as treating RBCs with dithiothreitol (DTT) or proteolytic enzymes (e.g., trypsin or papain), can reduce daratumumab‐related interference. However, these methods also destroy clinically significant antigens, thereby not allowing the detection of relevant alloantibodies, and are often restricted to reference laboratories because of their technical complexity and validation requirements [13, 19, 20, 21]. More practical alternatives, including CD38‐masking reagents or soluble CD38 (sCD38), have shown variable effectiveness and may still compromise alloantibody detection [22, 23].

Anti‐idiotypic antibodies directed against daratumumab offer a promising solution [24, 25]. A novel anti‐idiotypic reagent (DaraClear) was evaluated in an international multicentre study of 28 laboratories to assess its ability to neutralize daratumumab interference, preserve alloantibody detection and integrate into routine transfusion workflows.

This study provides the results of a comprehensive evaluation of DaraClear, a recombinant high‐affinity inhibitory anti‐idiotypic anti‐daratumumab reagent used as Fab fragment, across diverse laboratory settings, addressing a critical unmet need in transfusion medicine for patients receiving daratumumab therapy.

## MATERIALS AND METHODS

### Study design

**FIGURE 1:**
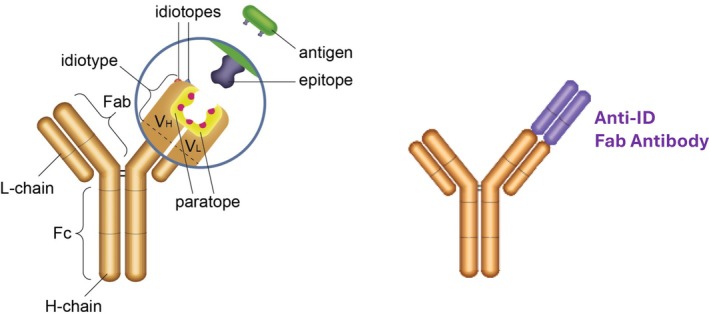
Mode of action of the anti‐idiotypic anti‐daratumumab. Daratumumab = IgG1κ monoclonal antibody targeting CD38. On the left‐hand side, the orange IgG represents daratumumab, an IgG1κ monoclonal antibody that binds CD38 on cells. The anti‐idiotypic reagent (purple, shown on the right‐hand side), used as a Fab fragment, binds specifically to the antigen‐binding site (paratope) of circulating daratumumab. This prevents daratumumab from binding CD38 on red blood cells (RBCs), thereby neutralizing its interference in serological testing.

This non‐interventional, multicentre study evaluated a novel anti‐idiotypic anti‐daratumumab (Figure 1) reagent across 28 transfusion laboratories in 15 countries (Table 1). Each laboratory received ready‐to‐use reagent and tested plasma samples containing daratumumab as part of routine pre‐transfusion workflows. Comparative scenarios, including parallel testing with other mitigation strategies such as DTT‐treated and enzyme‐treated RBCs, DaraEx (inno‐train Diagnostik GmbH) and sCD38 (Diagnostic Grifols, S.A.), were included. Institutional Review Board (IRB) or equivalent ethics approval was obtained where required. The study was conducted between December 2023 and November 2025, with all laboratory data prospectively collected and centrally analysed.

**TABLE 1:** Participating laboratories and countries in the anti‐idiotypic multicentre study.

Country	Number of sites
Argentina	1
Bulgaria	1
Finland	1
Italy	3
Malaysia	1
Norway	2
Poland	5
Spain	1
Slovenia	1
Sweden	1
Switzerland	3
Netherlands	1
Türkiye	2
United Kingdom	2
United States of America	3
15 countries	28 sites

*Note*: List includes all laboratories that participated in the multicentre study. The number of sites is indicated for each country.

### Samples

A total of 443 daratumumab‐containing plasma samples were tested, including both fresh and frozen specimens. All samples were confirmed as pan reactive by IAT prior to inclusion.

To assess interference with RBC antibody detection, mostly plasma from alloimmunized patients but also some commercial standards and typing reagents were used, representing 197 clinically diverse RBC antibodies (Table 2). A subset of plasma samples containing alloantibodies was pre‐diluted and then mixed with plasma containing daratumumab to simulate low‐titre (≤4) or weak (≤2) reactivity in the IAT gel card. Another subset was generated either by mixing plasma containing alloantibodies or by spiking commercial antisera into daratumumab‐containing plasma. Of the 197 samples included in the study, 191 contained both RBC antibodies and daratumumab, while 6 samples diluted with plasma without daratumumab were used to assess potential non‐specific effects of the anti‐daratumumab reagent. Seventeen samples were spiked with daratumumab (10–400 μg/mL), and the remaining were obtained from leftover plasma of daratumumab‐treated patients. All samples were evaluated by IAT to confirm pan reactivity with reagent RBCs.

**TABLE 2:** Red blood cell antibodies tested in daratumumab neutralization.

Specificities	Count
Anti‐D	26
Anti‐C	8
Anti‐c	9
Anti‐E	18
Anti‐e	4
Anti‐C^w^	4
Anti‐K	32
Anti‐Kp^a^	2
Anti‐Fy^a^	25
Anti‐Fy^b^	8
Anti‐Jk^a^	11
Anti‐Jk^b^	4
Anti‐S	3
Anti‐s	3
Anti‐M	22
Anti‐N	1
Anti‐Le^b^	2
Anti‐Lu^a^	2
Anti‐Lu^b^	2
Anti‐Yt^a^	1
Anti‐D + C	1
Anti‐c + K	1
Anti‐E + Mi^a^	1
Anti‐E + Jk^a^	1
Anti‐A	1
Anti‐B	2
Anti‐K + Kp^a^	1
Anti‐Vel	1
Anti‐MER2	1
Total	197

*Note*: The table lists all clinically significant RBC antibodies evaluated for interference by daratumumab and tested for neutralization using the anti‐idiotypic reagent.

Abbreviation: RBC, red blood cell.

### Anti‐idiotypic anti‐daratumumab

Anti‐daratumumab antibodies were selected from the HuCAL PLATINUM phage display library [26] through three rounds of panning on immobilized daratumumab, following the approach described by Jarutat et al. [27]. The library was blocked with normal human serum and an isotype‐matched human antibody before selection. Phage outputs were subcloned into a vector expressing monovalent Fab fragments, transformed into *Escherichia coli* TG1 and screened by enzyme‐linked immunosorbent assay (ELISA) for specific daratumumab binding. Positive clones were ranked by *k*_off_ values using bio‐layer interferometry [28], sequenced and expressed. Unique antibodies were purified via His‐tag affinity chromatography and characterized further. The highest affinity clone with the best inhibition ELISA performance was selected as the DaraClear reagent.

### Testing protocols

All laboratories performed neutralization tests in IAT using Bio‐Rad gel cards containing antihuman globulin (AHG) and either Bio‐Rad reagent RBCs or locally produced RBC panels. Some laboratories additionally used alternative column agglutination technology (CAT) methods. All testing was performed manually.

**FIGURE 2:**
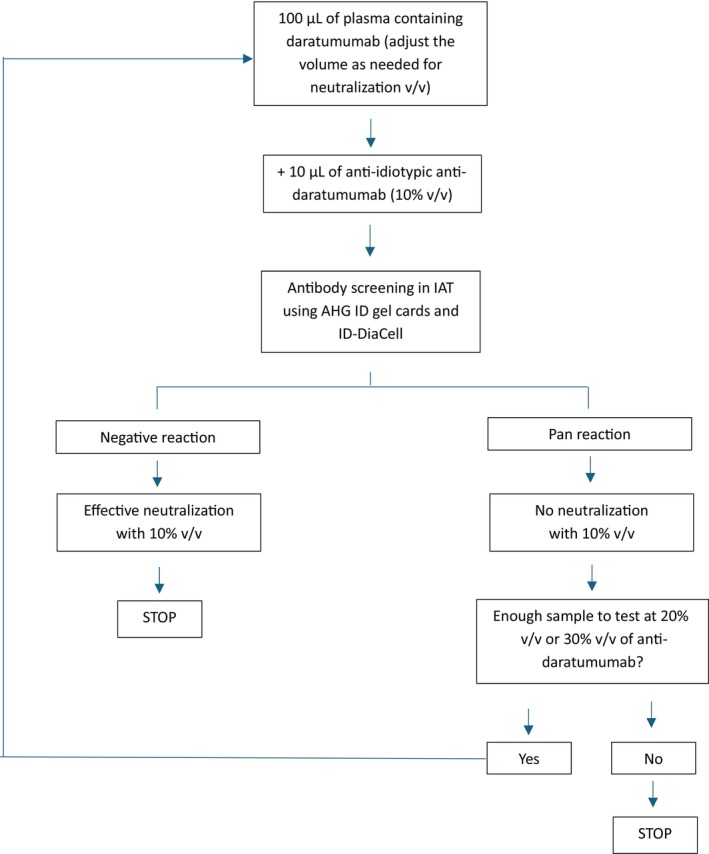
Testing protocol using the anti‐idiotypic anti‐daratumumab. Flowchart of the testing protocol (10%) using the anti‐idiotypic anti‐daratumumab reagent. The protocol outlines sample preparation, daratumumab neutralization and subsequent red blood cell (RBC) antibody detection steps. AHG, antihuman globulin; IAT, indirect antiglobulin test.

The anti‐daratumumab reagent was supplied ready to use. Plasma samples were initially treated with 10% (v/v) reagent. If interference persisted, concentrations were increased to 20%, and if necessary, to 30%. Treated plasma was tested by dispensing 25 μL of plasma onto 50 μL of reagent RBCs in AHG gel cards, followed by incubation for 15 min at 37°C and centrifugation for 10 min. Reaction strengths were recorded according to standard laboratory practice (Figure 2).

Three laboratories also evaluated an ‘add‐on’ protocol, adding 2.5 μL reagent directly to the ID‐Card with 25 μL plasma and 50 μL RBCs.

Parallel evaluations included RBCs treated with 0.2 M DTT or, for a small subset of samples, direct addition of DTT (0.04 M) to the RBC within the gel card before plasma addition [29, 30]. Two‐stage enzyme‐treated RBCs (trypsin in IAT gel cards and papain in neutral gel cards), DaraEx and sCD38 in IAT gel cards were also assessed where applicable [29]. Cross‐match testing, daratumumab titration in IAT gel cards and antibody class determination were performed at selected sites.

### Study outcomes

The primary objective was to assess the neutralization efficiency of this new anti‐idiotypic reagent in daratumumab‐containing samples at 10%, 20% and 30% concentrations. Secondary objectives included evaluating its impact on the detection of weak underlying RBC antibodies as well as assessing the ease of use and integration into routine transfusion laboratory workflows.

### Statistical analysis

Data analyses, including counts and sums, were performed using Microsoft Excel (Microsoft, Redmond, WA, USA). Ninety‐five percent confidence intervals (95% CIs) were calculated using the Diagnostic Statistics CI Calculator (https://www2.ccrb.cuhk.edu.hk/stat/confidence%20interval/Diagnostic%20Statistic.htm#Help). Differences in neutralization rates between products were evaluated using Fisher's exact test via the GraphPad online calculator (https://www.graphpad.com/quickcalcs/contingency2/).

## RESULTS

### Neutralization of daratumumab

Out of 443 daratumumab‐containing samples, 96.4% were neutralized (95% CI: 94.7–98.1). Of the 16 samples that were not neutralized, one was likely due to incomplete mixing with the anti‐daratumumab reagent, one was suspected to involve an autoantibody in a patient with AIHA and 14 samples could not be tested at a higher dose of either 20% or 30% because no additional reagent was available. Excluding these 14 samples where the full protocol could not be applied, the neutralization efficiency resulted in 99.5% (95% CI: 98.9–100). Neutralization was also successfully achieved using other CAT supports.

**FIGURE 3:**
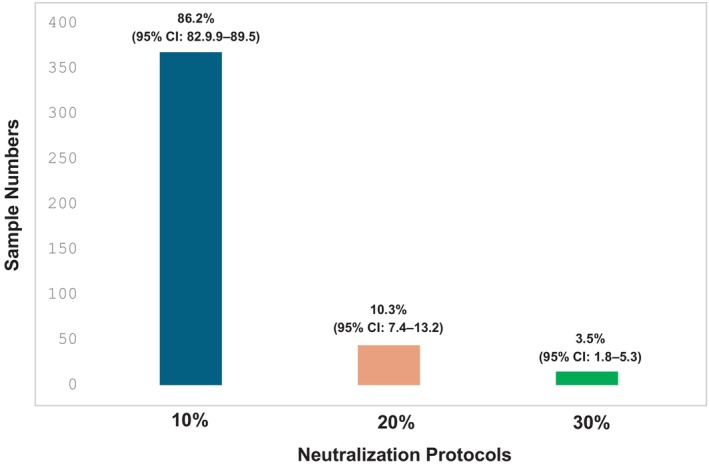
Neutralization efficiency of the anti‐idiotypic anti‐daratumumab across the 10%, 20% and 30% protocols. Neutralization efficiency of the anti‐idiotypic anti‐daratumumab reagent across 10%, 20% and 30% reagent protocols. Efficiency is expressed as the percentage of samples in which daratumumab interference was successfully neutralized. CI, confidence interval.

Among the 427 fully neutralized samples, 86.2% were neutralized using the 10% protocol (95% CI: 82.9–89.5), 10.3% with the 20% protocol (95% CI: 7.4–13.2) and 3.5% (95% CI: 1.8–5.3) with the 30% protocol (Figure 3).

In the add‐on protocol, neutralization efficiency was comparable (89% at 10%; *p* = 1.00, Fisher's exact test), indicating no statistically significant difference from the standard protocol using a plasma aliquot when 2.5 μL of DaraClear was dispensed prior to 25 μL of plasma.

### Comparison with other neutralization methods

Of the 210 samples tested using DTT‐treated RBCs, 93.3% were successfully neutralized (95% CI: 90.0–96.7), which was significantly lower than the neutralization rate observed with the anti‐daratumumab reagent (*p* < 0.0001, Fisher's exact test).

Among the 119 samples tested with trypsin‐ or papain‐treated RBCs, 84.9% were neutralized (95% CI: 78.4–91.3), also showing a statistically significant difference compared with the anti‐daratumumab reagent (*p* < 0.0001).

For 38 samples tested with DaraEx, 68.4% were neutralized (95% CI: 53.6–83.2), again demonstrating a statistically significant difference versus the anti‐daratumumab reagent (*p* < 0.0001).

Finally, all three samples tested with sCD38 were neutralized; statistical comparisons were not performed because of the small sample size (Table 3).

**TABLE 3:** Comparison of neutralization efficiency between anti‐idiotypic anti‐daratumumab, dithiothreitol, proteolytic enzymes and DaraEx.

	Anti‐daratumumab	DTT	Proteolytic enzymes	DaraEx
Daratumumab neutralization % (95% CI)	99.5 (98.9–100%)	93.3* (90.0–96.7)	84.9* (78.4–91.3)	68.4* (53.6–83.2)

*Note*: DTT: 0.2 M dithiothreitol used in 91.4% of samples; 0.04 M used as an add‐on in 8.6% of samples; proteolytic enzymes are papain or trypsin; DaraEx is a commercial daratumumab neutralization reagent. Neutralization efficiency is expressed as the percentage of samples in which daratumumab interference was successfully resolved.

Abbreviations: CI, confidence interval; DTT, dithiothreitol.

* *p* < 0.0001 (Fisher's exact test).

### Stability and cross‐match testing

All 10 samples remained fully neutralized when retested 1 h after treatment, as did all 17 samples retested 3 days after neutralization. Crossmatch testing was successfully resolved in 40 daratumumab‐containing samples, with a success rate of 90% using the 10% protocol (95% CI: 80.7–99.3) and 10% using the 20% protocol (95% CI: 7.0–19.3).

### Daratumumab titration study

A daratumumab titration study was conducted across five sites to evaluate the neutralization efficiency of three treatment protocols (10%, 20% and 30%) relative to anti‐CD38 titres. All sites used Bio‐Rad ID‐Cards; however, due to inter‐site differences in dilution process, buffers, RBC selection and titre interpretation, only data from sites 1 and 2 could be pooled. In contrast, sites 3, 4 and 5 yielded variable daratumumab titres, and their results were therefore analysed separately (Table 4).

**TABLE 4:** Inter‐site variation in daratumumab titration methodologies.

Site	Pre‐dilution	Serial dilution	Dilution medium	Dilution range	RBCs	Criteria for cell selection	Endpoint definition
1 and 2	None	Two‐fold	ID‐titration solution	1:1–1:32,000	ID‐DiaCell I (DiaCell I–III kit)	Same cell used for sites 1 and 2	1+
3	1:5	Two‐fold	Labex Diluent	1:10–1:5120	Cell from in‐house panel	No specific selection criteria	1+
4	None	Two‐fold	Isotonic wash solution	1:1–1:2048	ID‐DiaCell II (DiaCell I–III kit)	Strongest reactivity with anti‐CD38	1+
5	None	Two‐fold	ID‐Diluent 2	1:1–1:262,144	One cell from the DiaCell I–III kit	Strongest reactivity with anti‐CD38	Weakest positive

*Note*: Overview of inter‐site variability in daratumumab titration protocols, including pre‐dilution, dilution medium, dilution range, RBC reagents and endpoint definition.

Abbreviation: RBC, red blood cell.

Across sites 1 and 2, 90 samples were titrated. Neutralization was achieved in 83.3% of samples using the 10% protocol (95% CI: 75.6–91.0), 12.2% using the 20% protocol (95% CI: 5.4–19.0) and 4.4% using the 30% protocol (95% CI: 0.2–8.7).

Using the 10% protocol, all samples with daratumumab titres ≤1024 (44% of samples) were neutralized. Neutralization rates decreased with increasing titre, falling to 92.9% at a titre of 2048 (16%), 82.6% at 4096 (26%), 30.0% at 8192 (11%) and 0% at 16384 (3%).

With the 20% protocol, complete neutralization was achieved for all samples with titres ≤4096 (86% of samples), as well as for 71.4% of samples with a titre of 8192 and 33.3% with a titre of 16,384. The remaining 4% of samples required the 30% protocol to achieve neutralization (Table 5).

**TABLE 5:** Sites 1 and 2: Anti‐CD38 titration study compared with neutralization protocol.

Protocol	Daratumumab titres (% of samples)				
	≤1024 (44%)	2048 (16%)	4096 (26%)	8192 (11%)	16,384 (3%)
10%	100% (95% CI: 100–100)	92.9% (95% CI: 79.4–100)	82.6% (95% CI: 67.1–98.1)	30% (95% CI: 1.6–58.4)	0% (95% CI: 0–0)
20%	NA	100% (95% CI: 100–100)	100% (95% CI: 100–100)	71.4% (95% CI: 38–100)	33.3% (95% CI: 0–86.7)
30%	NT	NT	NT	100% (95% CI: 100–100)	100% (95% CI: 100–100)

Abbreviation: CI, confidence interval; NT, not tested.

At Site 3, a total of 10 samples were included, with 5 known to be neutralized at 10% and 5 at 20%. All samples with titres between 160 and 320 were fully neutralized at 10%. In contrast, among samples with higher titres (2560–5120), only 16.7% were neutralized at 10%, with the remaining samples requiring escalation to the 20% protocol (Table 6).

**TABLE 6:** Site 3: Anti‐CD38 titration study compared with neutralization protocol.

Site 3	Daratumumab titres (% of samples)	
Protocol	160–320	2560–5120
10%	100% (95% CI: 100–100)	16.7% (95% CI: 0–46.5)
20%	NT	100% (95% CI: 100–100)
30%	NT	NT

Abbreviation: CI, confidence interval; NT, not tested.

Fifteen samples were analysed at Site 4. Overall, 86.7% were neutralized using the 10% protocol. This included 92.9% of samples with titres ≤1024, which accounted for 93% of the cohort. The remaining samples, all with a titre of 2048 (7%), were fully neutralized using the 20% protocol (Table 7).

**TABLE 7:** Site 4: Anti‐CD38 titration study compared with neutralization protocol.

Site 4	Daratumumab titres (% of samples)	
Protocol	≤1024 (93%)	2048 (7%)
10%	92.9% (95% CI: 79.4–100)	0% (95% CI: 0–0)
20%	100% (95% CI: 100–100)	100% (95% CI: 100–100)
30%	NT	NT

Abbreviation: CI, confidence interval; NT, not tested.

At Site 5, 42 samples were titrated, with 90.5% neutralized using the 10% protocol. All samples with titres ≤16,384 (83% of samples) were neutralized at 10%, as were 50.0% of samples with titres between 16,384 and 65,536 (15%). The single sample with a titre of 262,144 required the 20% protocol for complete neutralization (Table 8).

**TABLE 8:** Site 5: Anti‐CD38 titration study compared with neutralization protocol.

Site 5	Daratumumab titres (% of samples)			
Protocol	≤16,384 (83%)	32,768 (10%)	65,536 (5%)	≥131,072 (2%)
10%	100% (95% CI: 100–100)	50% (95% CI: 1.0–99.0)	50% (95% CI: 0–100.0)	0% (95% CI: 0–0)
20%	NT	100% (95% CI: 100–100)	100% (95% CI: 100–100)	100% (95% CI: 100–100)
30%	NT	NT	NT	NT

*Note*: Anti‐CD38 titration results are shown for each site (sites 1–5) and compared with corresponding standard neutralization protocols. Neutralization efficiency is expressed as the percentage of samples in which daratumumab interference was completely resolved. Sites refer to participating laboratories in the multicentre study.

Abbreviation: CI, confidence interval; NT, not tested.

### Detection of RBC antibodies

Among the 191 samples containing both daratumumab and RBC antibodies, 98.4% were successfully neutralized (95% CI: 96.7–100). Of the five samples that were not neutralized, four were not tested at 20% due to insufficient anti‐daratumumab volume, and one was not neutralized at 20% and was not tested at 30%.

**FIGURE 4:**
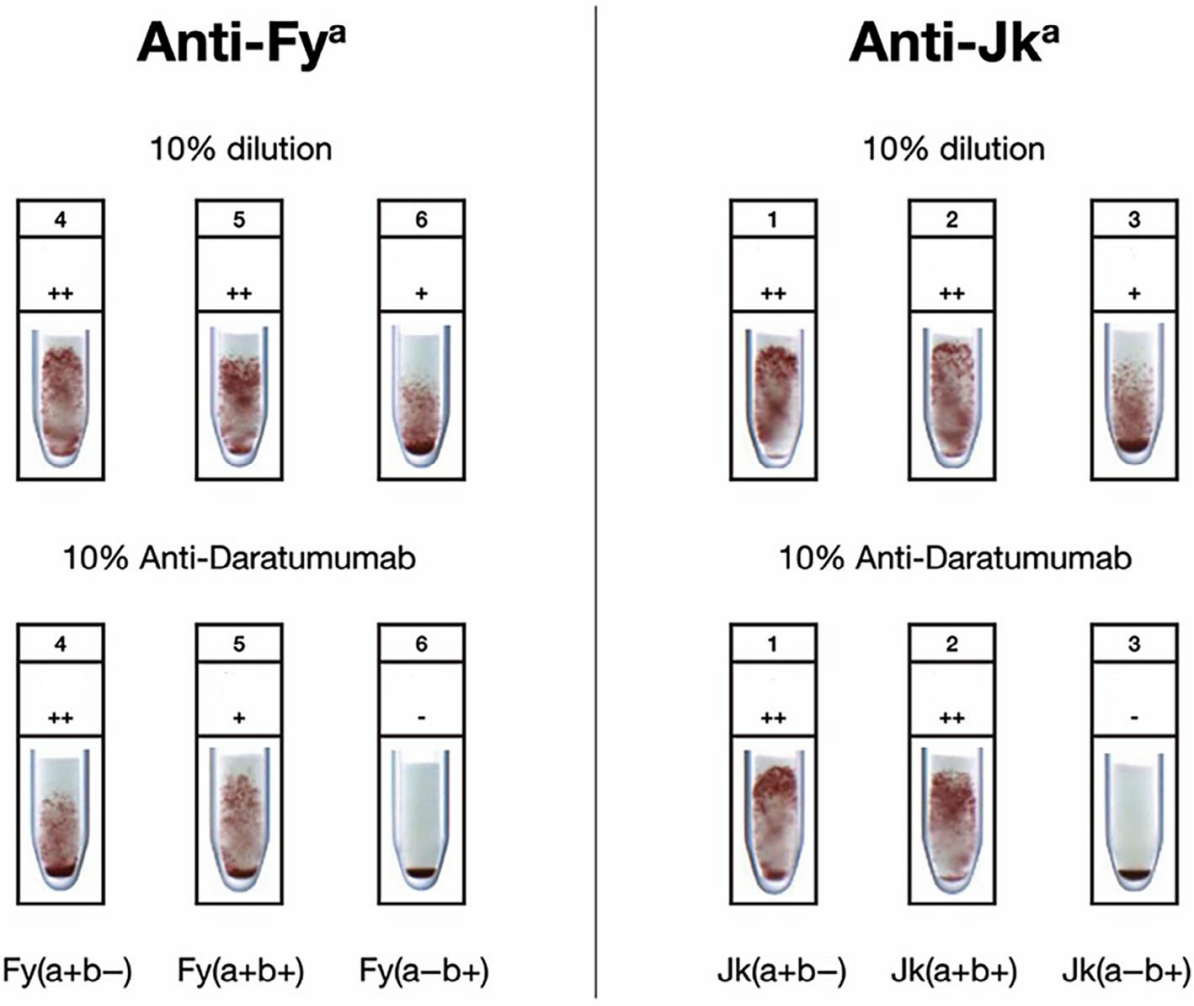
Neutralization tests with weak anti‐Fy^a^ and anti‐Jk^a^. Two samples containing weak anti‐Fy^a^ and anti‐Jk^a^ are shown. The 10% dilution control demonstrates pan reactivity with antigen‐positive and antigen‐negative red blood cells. Neutralization with 10% anti‐daratumumab allows detection of weak antibodies, with reactivity restricted to antigen‐positive cells (single‐ and double‐dose) and no reactivity with antigen‐negative cells.

Following neutralization, 96.4% of 197 RBC antibodies (95% CI: 93.9–99) were detectable (Figure 4). The seven undetectable antibodies included three anti‐M, two anti‐Le^b^ and two weak anti‐Jk^b^ antibodies.

Weak anti‐Jkᵇ antibodies were evaluated using locally produced RBC panels within the same laboratory. Of the three anti‐Jkᵇ samples, two were not detected following neutralization but exhibited very weak reactivity with DTT‐treated cells, while the third remained weakly detectable after treatment with the anti‐idiotypic reagent. As diluted anti‐Jkᵇ samples were not screened prior to neutralization, it remains unclear whether these antibodies were detectable before treatment when tested with DTT‐untreated cells.

All three undetectable anti‐M samples originated from a single site and were immunoglobulin M (IgM), whereas a concurrently tested anti‐M antibody from the same laboratory was immunoglobulin G (IgG) and remained detectable. To further investigate this apparent IgM‐specific non‐detection, 11 anti‐M samples were selected, isotype‐characterized using DTT (6 IgG and 5 IgM) and combined with daratumumab‐containing plasma. All anti‐M antibodies were detectable regardless of the immunoglobulin class.

## DISCUSSION

To our knowledge, this multicentre study provides one of the most comprehensive evaluations to date of a reagent designed to neutralize daratumumab interference in pre‐transfusion testing. Although daratumumab has transformed the treatment of MM, it remains a substantial challenge for transfusion laboratories because of its pan reactivity in IAT. This interference can mask clinically significant alloantibodies, delay transfusion support and increase laboratory workload. The present findings demonstrate that the anti‐daratumumab reagent (DaraClear) offers an effective, scalable and workflow‐compatible solution to these challenges.

Neutralization of daratumumab interference was achieved in 99.5% of samples, with most cases (86.2%) resolved using the 10% protocol. This performance was significantly superior to DTT and trypsin/papain (*p* < 0.0001), which neutralized 93.3% and 84.9% of tested samples, respectively, and are associated with considerable limitations, including destruction of clinically significant blood group antigens and increased labour requirements. Neutralization was also significantly superior to DaraEx, which achieved a 68.4% success rate (*p* < 0.0001).

The small number of initially unresolved samples was likely attributable to pre‐analytical factors or the presence of underlying autoantibodies rather than intrinsic limitations of the reagent. Importantly, escalation of the reagent concentration to 20% or 30% resolved interference in all samples with high daratumumab titres, confirming a concentration‐dependent neutralization effect. Titration studies conducted across multiple sites consistently demonstrated that while the 10% protocol was sufficient for most clinical samples, higher concentrations might be required in rare cases with very high daratumumab levels. These findings support a flexible, stepwise approach adaptable to routine laboratory practice.

Preservation of clinically significant alloantibody detection is essential for any daratumumab mitigation strategy. Across 197 alloantibody‐containing samples, the reagent preserved detection in 96.4% of antibodies while sustaining effective neutralization. This represents a high level of clinical safety and compares favourably with alternative methods such as DTT or enzyme treatment [20], both of which may destroy clinically significant blood group antigens and sCD38, which has been shown to neutralize antibodies to antigens of the FY (Duffy) blood group system [22].

The few antibodies not detected after neutralization were predominantly specificities generally considered of limited clinical significance, including anti‐M and Lewis antibodies. For the very weak anti‐Jkᵇ antibodies, factors such as sample preparation, dilution and differences in red cell panels likely influenced reactivity, as other samples with identical specificities were detected at different sites. Confirmatory DTT testing further demonstrated that both IgG and IgM anti‐M antibodies remained fully detectable under standardized conditions following neutralization. Overall, these findings indicate that the anti‐idiotypic reagent effectively preserves detection of clinically relevant antibodies.

A major strength of this study is the inclusion of 28 laboratories across 15 countries, encompassing a wide diversity of workflows, expertise and testing environments. Despite this heterogeneity, neutralization performance remained consistently high. Evaluation of an add‐on protocol at three sites further demonstrated compatibility with existing column agglutination workflows, providing additional operational flexibility. Unlike DTT or proteolytic enzyme methods, which require red cell modification, technical expertise and careful antigen selection, the anti‐idiotypic reagent is simple to use, requires no red cell manipulation and integrates seamlessly into routine laboratory practice. Stability data further support its practicality, with sustained neutralization over time.

The ability to neutralize daratumumab interference while preserving alloantibody detection addresses a major unmet need in transfusion support for treated patients. Current strategies frequently rely on phenotype‐ or genotype‐matched red cell units, despite evidence that alloimmunization rates in daratumumab‐treated patients are relatively low compared with other chronically transfused populations. Availability of a reliable neutralization reagent may allow laboratories to return to standard serological testing, reducing reliance on extended matching, improving turnaround times, lowering workload and reagent costs and enabling more targeted allocation of extended‐typed units to patients at highest alloimmunization risk. This approach aligns with the broader shift towards precision transfusion support that balances safety, efficiency and resource stewardship.

This study represents the largest multicentre evaluation of a daratumumab neutralization reagent to date, enhancing the generalizability of the findings. Inclusion of a broad spectrum of alloantibodies, including weak and low‐titre clinically significant antibodies, allowed a robust assessment of reagent performance. Direct comparisons with established mitigation strategies and evaluation across variable daratumumab concentrations further support the reagent's practical applicability. Limitations include incomplete testing at all reagent concentrations for a small number of samples, limited comparisons with sCD38 and the absence of clinical outcome or post‐transfusion follow‐up data.

In conclusion, the anti‐idiotypic anti‐daratumumab reagent evaluated in this study provides an effective and practical solution to daratumumab interference in pre‐transfusion testing. It reliably neutralizes daratumumab, preserves clinically significant alloantibody detection and integrates smoothly into routine laboratory workflows across diverse settings. Compared with existing mitigation strategies, this approach demonstrates improved safety and efficiency and may facilitate a return to standard pre‐transfusion testing practices in this expanding patient population.

## CONFLICT OF INTEREST STATEMENT

A.R., M.R. and A.K. are employees of Bio‐Rad Laboratories. M.R. and A.K. are developers of the anti‐idiotypic anti‐daratumumab reagent (DaraClear) and A.R. served as the corresponding author. All other co‐authors are affiliated with independent laboratories and have declared no conflicts of interest. The study was conducted collaboratively between Bio‐Rad Laboratories and participating laboratories to ensure scientific rigour and transparency.

## Data Availability

The data that support the findings of this study are available from the corresponding author upon reasonable request.
